# Recognition of Connective Tissue Disease-Related Interstitial Pneumonia Based on Histological Score—A Validation Study of an Online Diagnostic Decision Support Tool

**DOI:** 10.3390/diagnostics11081359

**Published:** 2021-07-28

**Authors:** Mutsumi Ozasa, Yoshiaki Zaizen, Kazuhiro Tabata, Kensuke Kataoka, Shuntaro Sato, Andrey Bychkov, Noriho Sakamoto, Hiroshi Mukae, Yasuhiro Kondoh, Junya Fukuoka

**Affiliations:** 1 Department of Pathology, Nagasaki University Graduate School of Biomedical Sciences, Nagasaki 852-8501, Japan; 0717mutumi@gmail.com (M.O.); zaizen_yoshiaki@med.kurume-u.ac.jp (Y.Z.); tabatak.kufm@gmail.com (K.T.); bychkov.andrey@kameda.jp (A.B.); 2 Department of Respiratory Medicine, Nagasaki University Graduate School of Biomedical Sciences, Nagasaki 852-8501, Japan; nsakamot@nagasaki-u.ac.jp (N.S.); hmukae@nagasaki-u.ac.jp (H.M.); 3 Department of Respiratory Medicine and Allergy, Tosei General Hospital, Seto 489-8642, Japan; kataoka@tosei.or.jp (K.K.); konyasu2003@yahoo.co.jp (Y.K.); 4 Clinical Research Center, Nagasaki University Hospital, Nagasaki 852-8501, Japan; shuntarosato@nagasaki-u.ac.jp; 5 Department of Pathology, Kameda Medical Center, Kamogawa 296-8602, Japan

**Keywords:** idiopathic pulmonary fibrosis, connective tissue disease, lung disease, interstitial, histology, pathologists

## Abstract

Objectives: to evaluate the number of cases of idiopathic pulmonary fibrosis (IPF) that included histological features of connective tissue disease (CTD) and to check whether they demonstrated the clinical features of CTD, using a previously reported CTD-interstitial pneumonia (IP) index that histologically differentiates CTD-associated and idiopathic IP. Methods: patients diagnosed with IPF following video-assisted thoracoscopic biopsy through multidisciplinary team diagnosis between 2014 and 2017 were selected. Pathological observation was made by four pathologists who scored eight observational items needed for the CTD-IP index. Cases determined as CTD, by the CTD-IP index, were extracted, and their clinical features were compared. Results: a total of 94 cases of IPF were identified, of which 20 were classified into the CTD group using the CTD-IP index with reasonable interobserver agreement (k = 0.76). Cases pathologically classified into the CTD group were significantly associated with female sex, non-smoking history, autoantibody positivity, and CTD symptoms (*p* = 0.01, 0.03, 0.01, and 0.04, respectively). Conclusions: patients with IPF with pathological findings of CTD showed clinical characteristics similar to those of patients with CTD.

## 1. Introduction

Idiopathic interstitial pneumonias (IIPs) are interstitial pneumonias (IPs) that have no clear systemic disease or cause; they are classified into various types based on the clinical, radiologic, and pathological findings [1,2,3]. Among the different types of IIPs, idiopathic pulmonary fibrosis (IPF) has the worst prognosis. Although there is no definitive treatment yet available, antifibrotic drugs are considered a promising, innovative treatment [4]. The use of anti-inflammatory drugs, such as corticosteroids, N-acetylcysteine, and immunosuppressants is discouraged in IPF due to the associated worsened outcomes and a high risk of adverse events [5,6].

However, there are a number of IPF cases where clinical and pathological findings are suggestive of connective tissue disease (CTD) [7,8,9]. Such cases are constantly debated in multidisciplinary discussions (MDD) for inconsistency in the interpretation of histological diagnosis among pathologists. For instance, some MDD teams may diagnose the usual IP (UIP) cases with CTD features as unclassifiable IIP [10,11].

Lung-dominant CTD [12] and undifferentiated CTD [13] are the terms proposed for the cases that present clinical or pathological features of CTD but where a definitive diagnosis of CTD cannot be made. Hence, to help define the disease more uniformly, a working group of the American Thoracic Society/European Respiratory Society proposed IP with autoimmune features (IPAF) as a provisional research category [14]. While multiple findings, such as clinical, serologic, and morphologic domains are included in the criteria for IPAF, most pathological variables are selected based on expert opinions, with no clear evidence of independent or confounding variables. Previous reports have documented pathological findings, other than those indicated in IPAF or lung-dominant-CTD criteria, as indicative of CTD [8,15], suggesting that different standards have been used to histologically determine CTD-associated IPs.

In view of this, a detailed histopathological scoring of 154 cases of IP was conducted to identify the parameters that reliably differentiated CTD-IPs and IIPs. Furthermore, a CTD-IP index capable of distinguishing these two disease groups was formulated; the formula was used to create a user-friendly application (www.CTDIP.com accessed on 17 September 2020), in which the user could enter a histological score for the parameter, and the application would yield the calculated result [16].

Using this CTD-IP index designed to distinguish between IIPs and CTD-IPs, two out of 32 cases in our study cohort, originally diagnosed as IIP, were found to be CTD-IPs. The extent of CTD-like clinical features displayed in IIP-diagnosed cases, with histological characteristics of CTD-IPs, is yet to be revealed. In cases with pathological patterns of UIP, depending on whether they are diagnosed as IPF or CTD-related UIP, there are considerable differences in the treatment. Therefore, distinguishing the two disease entities is critical and clinically significant. In cases where software assessment of CTD-IP showed clinical features of CTD and the histological events inside the lung reflected systemic inflammation in the spectrum of CTD, different treatment strategies should be considered.

The aim of this study was to evaluate whether the CTD-IP index could reproducibly identify and assort a group of cases harboring autoimmune features of IPF.

## 2. Materials and Methods

This study was approved by the Nagasaki University Hospital Clinical Research Ethics Committee (protocol no: 20101918); an informed consent was obtained from all patients. All methods were carried out in accordance with the relevant guidelines and regulations.

### 2.1. Case Selection

Cases determined as IPF on video-assisted thoracoscopy (VATS) biopsy, between 2014 and 2017, were enrolled. The cases categorized as unclassifiable IIP through MDD were included; the cases showed UIP patterns on the histopathological investigation, and were given a working diagnosis of IPF. Cases with definite CTD, and cases subsequent to hypersensitivity pneumonitis and pneumoconiosis were excluded from the study. All hematoxylin and eosin (HE) slides were digitized at 200× using an Aperio CS2 slide scanner (Leica Biosystems Imaging, Vista, CA, USA). Biopsies were obtained from two or three lobes, and all the slides were included in the study. The slides were evaluated by four pathologists, including two expert pulmonary pathologists and two senior residents, who were blinded to the clinical data. Previously, eight histological parameters were reported that could differentiate CTD-IPs and IIPs accurately [14]. These included fibroblastic focus (FF), smooth muscle hyperplasia (SMH), cellular IP (CIP), dense perivascular collagen (DPVC), fat metaplasia (Fat), prominent plasmacytic infiltration (plasm), presence of lymphoid follicle with germinal center (LyGC), and airspace fibrin (AF). These parameters were evaluated for a score of 0 (none), 1 (mild), 2 (moderate), or 3 (marked).

Based on the scores, the previously reported CTD-IP index, indicated below, was used to identify the cases with features of CTD. The formula used to calculate the probabilities (*P*) based on the estimated values for each finding is as follows:

$$\begin{array}{l} P(Y=\mathrm{CTD-IP}\;\mathrm{Markers})=\exp(Z)/1+\exp(Z)\\ Z=+1.65-1.09(\mathrm{FF}\;\mathrm{score})-0.81(\mathrm{SMH}\;\mathrm{score})-0.85(\mathrm{CIP}\;\mathrm{score})-0.86(\mathrm{DPVC}\;\mathrm{score})\\ -0.57(\mathrm{Fat}\;\mathrm{score})+0.86(\mathrm{Plasm}\;\mathrm{score})+0.64(\mathrm{LyGC}\;\mathrm{score})+2.47(\mathrm{AFscore}) \end{array}$$

The probability was generated by allocating a score to each histopathological parameter. Cases with *P* > 0.5 were separated as CTD-IP using the software. Cases in which all the four evaluators gave a diagnosis of CTD-IP were classified to the CTD group, and the remaining cases were classified to the idiopathic group. Agreement rates of the evaluators on the software differentiation, as well as on each parameter, were examined. Clinical differences between the groups, according to the observation by each pathologist, were examined to determine whether the separation showed an identical trend. 

The clinical information collected were age, sex, smoking history, CTD symptoms (Raynaud’s phenomenon, joint pain/swelling, photosensitivity, weight loss, morning stiffness, dry mouth/eyes, dysphagia, gastroesophageal reflux, rash, oral ulceration, proximal muscle weakness, mechanic’s hand, ulceration of the fingertips, arthritis, palmar vascular dilatation, unexplainable edema of the fingers, Gottron’s sign), autoantibodies (anti-nuclear antibody [ANA], rheumatoid factor [RF], anti-Ro [SS-A], anti-La [SS-B], anti-topoisomerase [Scl-70], anti-tRNA synthetase, anti-dsDNA, anti-ssDNA, anti-CCP, anti-MDA5, proteinase-3-anti-nuetrophil cytoplasmic antibody [PR3-ANCA], myeloperoxidase [MPO]-ANCA, Krebs von den Lungen-6 [KL-6], surfactant protein D [SP-D]) %forced vital capacity (FVC), % diffusing capacity of carbon monoxide (DLco), bronchoalveolar lavage fluid (BALF) result, and arterial blood gas.

### 2.2. Statistical Analysis

Clinical observations were compared between the CTD and the idiopathic group. 

The chi-squared test and Fisher’s exact test were used to evaluate the association between the two groups and the clinical factors. A *p*-value of <0.05 was considered statistically significant. Agreement among evaluators was examined by calculating the κ coefficient. Statistical analysis was performed using EZR software [17]. 

## 3. Results

### 3.1. Histological Detection of CTD Group by the CTD-IP Index

Among the 206 cases with IP, 112 cases were excluded due to other histological types such as non-specific IP, definitive CTD, or other definitive diseases such as hypersensitivity pneumonitis and sarcoidosis. Eventually, 94 cases were included in the study, which had a UIP pattern and were diagnosed as IPF through MDD. Among these cases, using software, 20 were classified into the CTD group; the remaining 74 patients were classified into the idiopathic group. Among these 74 cases, 52 cases were classified into the idiopathic group by all four pathologists. The κ coefficient of diagnosis was 0.761.

Among the 22 cases classified into the idiopathic group, but not agreed upon by all four evaluators, only one of the four reviewers indicated a different result in 16 cases. Of these cases, 11 and five were determined as IIP and CTD, respectively, by the three evaluators. On a further breakdown of 16 cases, eight cases were classified based on FF scores. In cases with very few other observational findings, the presence or absence of FF could distinguish the CTD group from the idiopathic group.

**Figure 1:**
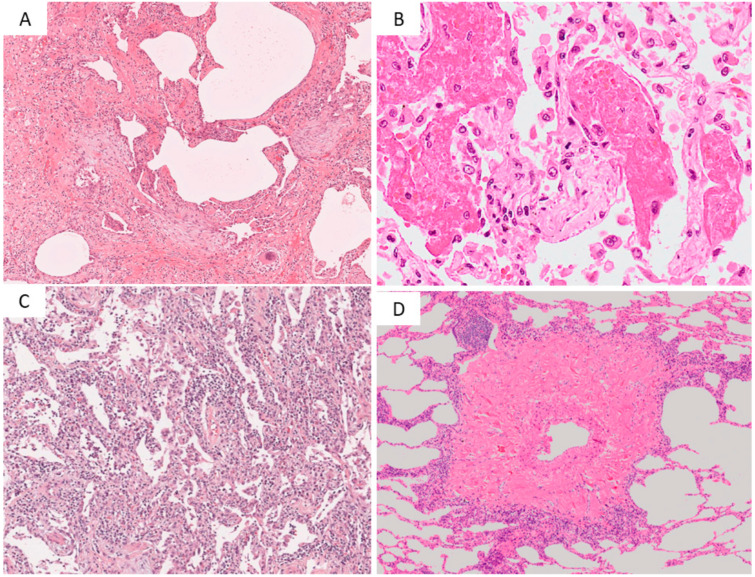
Pathological features. In the scoring items, fibroblastic focus ((**A**): HE, ×100) and airspace fibrin ((**B**): HE, ×100) showed a high concordance rate among the evaluators. On the contrary, cellular IP ((**C**): HE, ×50) and dense perivascular collagen ((**D**): HE, ×50) showed a low concordance rate among the evaluators. HE, hematoxylin and eosin.

The κ coefficients, for each observational item, were FF (κ = 0.532), SMH (κ = 0.512), CIP (κ = 0.327), DPVC (κ = 0.184), Fat (κ = 0.413), plasm (κ = 0.392), LyGC (κ = 0.49), and AF (κ = 0.602). FF and AF had a higher agreement rate, while CIP and DPVC showed considerable disagreement, suggesting that it was difficult to make an objective decision (Figure 1 and Table 1).

### 3.2. Correlation with Clinical Autoimmune Features

The clinical information of the CTD and idiopathic groups is presented in Table 2. There were a greater number of female and non-smoking patients in the CTD group than in the idiopathic group. In the CTD group, a greater number of patients with positive autoantibodies on blood examination and physical observations indicating CTD were observed. The ratio of cluster of differentiation 4 and 8 was the only variable that had a strong correlation with the idiopathic group. No significant differences were observed in KL-6, SP-D, respiratory function, or blood gas findings.

If the histological criteria suggested IPAF, LyGC ≥ 2, and plasm ≥ 2, were used for the categorization; there were only nine cases in which all the evaluators formulated a pathological diagnosis of IPAF. On correlating the clinical findings to the score given by each evaluator, the results were almost identical for all evaluators except one (Supplementary Table S1).

**Figure 2:**
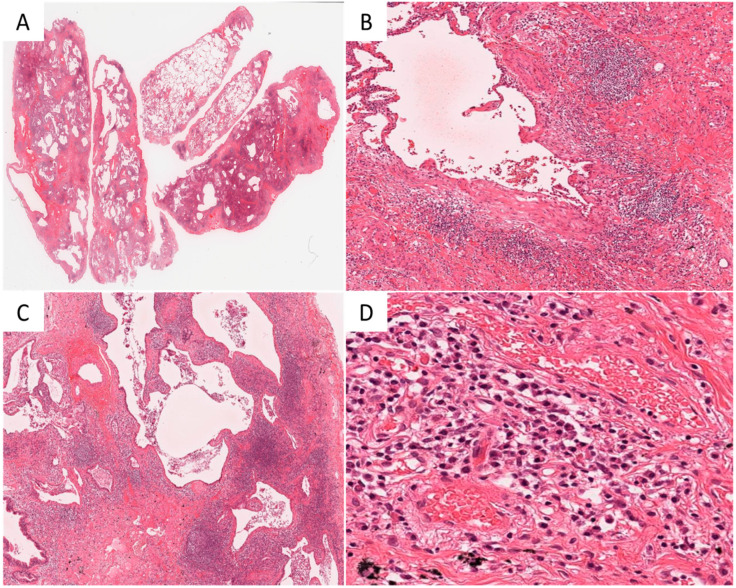
The case of idiopathic pulmonary fibrosis showing CTD-like histopathological features. (**A**): the whole image of VATS tissue shows a peripheral distribution and abrupt change. (HE, ×0.5). (**B**): dense fibrosis and fibroblastic focus suggesting UIP are seen. (HE, ×100). (**C**,**D**): a large number of lymphoid follicles with germinal centers and plasmacytic infiltration are found in the interstitium, indicating collagen disease. CTD, connective tissue disease; VATS, video-assisted thoracoscopy; UIP, usual interstitial pneumonia; HE, hematoxylin and eosin.

The pathological features of a representative case from the CTD group are shown in Figure 2. The pathological diagnosis of UIP was determined by the presence of dense fibrosis with architectural destruction and abrupt transition from the normal lung. The scores for the representative case are shown in Table 3. Plasm and LyGC were scored high (2 or 3) by all pathologists. The representative patient was a 68-year-old man who had no smoking history or symptoms related to CTD, but blood examination showed positive results with 128 IU/mL of RF. The patient was diagnosed with IPF through MDD due to failure to fulfill the criteria of CTD.

## 4. Discussion

The findings and CTD-IP index, for distinguishing CTD-IP and IIPs, had been reported previously. Patients diagnosed with IPF are excluded from anti-inflammatory treatments, such as steroids and immunosuppressants, and receive antifibrotic therapy. Here, cases without definite CTD, which were determined as IPF in the MDD and displayed pathological UIP patterns, were examined. On evaluation, 30% of the cases (20/94) were histologically assigned to the CTD group using the CTD-IP index. The comparison of clinical characteristics revealed a greater number of cases, with symptoms and/or autoantibodies suggestive of CTD, in females and non-smokers.

The results indicated that the eight histological parameters, utilized for the scoring, reflected the clinical and serological features of the CTD group; therefore, in patients, classified into the CTD group using the formula, treatment options similar to that for CTD, may be beneficial.

In recent years, the cases that did not fulfill the diagnostic criteria but exhibited clinical and imaging features similar to CTD, have been diversely termed as undifferentiated CTD or lung-dominant-CTD. In 2015, the American Thoracic Society/European Respiratory Society defined these cases as IPAF [14]. The classification of cases as IPAF requires the presence of IP on high-resolution computed tomography or surgical lung biopsy, non-fulfillment of the diagnostic criteria of CTD, and in accordance with at least two of the three domains: clinical, serological, and morphological.

While pathological observations are defined in the morphological domain, UIP is not included as a histological parameter. Moreover, it has been reported that 62% of cases with IIP, which indicated, but did not fulfill, the diagnostic criteria of CTD, showed UIP on high-resolution computed tomography [18]; the pathological UIP was seen in 73.5% of the cases diagnosed as IPAF [19]. Thus, indicating that a certain number of cases, showing features of CTD, are diagnosed as IPF because UIP is not included in the IPAF classification criteria [20]. Radiological images were not examined in this study. However, if LyGC and plasm scores of two or above were considered sufficient for the morphological domain, only nine cases were pathologically categorized as IPAF; meanwhile, by the software, 20 cases were pathologically categorized into the CTD group, suggesting that approximately half of the cases were not recognized under the IPAF criteria.

Additionally, inter-pathologist agreement rates, for PLSM and LyGC, considered the basis for determining IPAF, were moderate with kappa coefficients of approximately 0.4 to 0.5. Therefore, it could be speculated that a significant number of cases with CTD indications may remain unrecognized under the current IPAF criteria.

It still remains unclear whether distinguishing cases of IPF with CTD findings from IPF is beneficial for the patients or not. Although, treatment for CTD should be indicated as a primary choice in patients fulfilling the criteria of CTD during the follow-up; the patients followed up for IPF rarely develop CTD, even with clinical findings suggestive of CTD [21,22] and, thus, IPF treatment may be continued. The treatment response to corticosteroids and immunosuppressants as well as prognosis in cases with IPF with CTD findings is yet to be elucidated. Therefore, further clinical trials for establishing evidence on the association of CTD findings with treatment response are warranted.

Despite the low agreement rates of each score (κ = 0.1–0.5), diagnostic agreement using the software was high with a kappa coefficient of 0.76, implying that the use of the software derives analogous diagnostic results irrespective of the expertise of the evaluator as a pathologist. This agreement rate is higher than that of expert physicians in determining IPF; thus, emphasizing its effectiveness [23]. Considering the study results of Walsh et al., where the diagnostic agreement of CTD-IPs was low at kappa coefficient 0.22 [24], application of a highly objective CTD-IP index is very valuable.

To apply this study to clinical practice, clinical trials, preferably randomized control trials, are needed to compare steroid/immunosuppressants and anti-fibrotic treatments in patients with histological UIP and suggestive but not definitive CTD.

This study has several limitations. First, it was a retrospective study. Second, information on the prognosis and treatment effects were not obtained; therefore, the association between the use of CTD-IP index and clinical outcomes and prognosis could not be determined. Third, the data were limited to surgical lung biopsy and, thus, generalizability of this index to the cryobiopsies could not be determined. Moreover, cryobiopsies are seldom taken for pleural and subpleural tissues. In terms of strengths, this study was a first attempt to correlate pathological CTD-like findings in IPF with clinical symptoms and autoantibodies. With the use of this formula, it is possible to generate highly reproducible judgments regardless of investigator experience or specialty.

## 5. Conclusions

The study succeeds in the creation of a standardized approach for the pathological identification of cases with CTD-like symptoms and autoantibodies from IPF with high reproducibility. The previously reported CTD-IP index reproducibly found histologically presenting CTD cases among the IPF cases we studied, in agreement with the clinical and serological data.

## Figures and Tables

**Table 1:** Agreement of eight histological parameters.

High Agreement	Low Agreement
Airspace fibrin (κ = 0.602)	Dense perivascular collagen (κ = 0.184)
Fibroblastic focus (κ = 0.532)	Cellular IP (κ = 0.327)
Smooth muscle hyperplasia (κ = 0.512)	Plasmacytic infiltration (κ= 0.392)
Lymphoid follicle with germinal center (κ = 0.49)	Fat metaplasia (κ = 0.413)

**Table 2:** Patients’ distribution.

**Variable**	**CTD Group** **(*n* = 20)**	**Group IPF** **(*n* = 74)**	***p*-Value**
**Age**	60.9 ± 11	62.8 ± 7.08	0.37
**Sex**			0.01
Female	15	16	
Male	5	58	
**Smoking history**			0.03
Ex	4	53	
Never	16	21	
**CTD symptom**			0.04
positive	12	25	
negative	8	49	
**IPAF symptom**			0.35
positive	5	12	
negative	15	62	
**Autoantibody**			0.01
positive	16	34	
negative	4	40	
**IPAF serological domain**			<0.01
positive	13	14	
negative	7	60	
**Blood parameters**			
KL-6	1438 ± 1019	1830 ± 2433	0.48
SP-D	321.6 ± 235.9	297.6 ± 199.3	0.65
PaO_2_	83.4 ± 14.8	81 ± 14.6	
PaCO_2_	43.5 ± 10.5	41.7 ± 8.91	0.46
**Respiratory function**			
Low SPO_2_	88 ± 5.14	84.8 ± 8.81	0.14
%FVC	82.1 ± 19	86.5 ± 16.7	0.31
%Dlco	69.4 ± 13.9	73.2 ± 45.2	0.72
**BAL cell** **s**	2.71 ± 3.4	2.02 ± 1.3	0.12
MΦ	74.2 ± 22.6	75.1 ± 24.7	0.88
Ly	15.13 ± 18.1	11.3 ± 15.3	0.35
Neut	5.74 ± 12.8	8.09 ± 13.1	0.48
Eo	3.66 ± 3.8	3.32 ± 6.99	0.84
CD4/8	1.49 ± 1.33	2.89 ± 2.36	0.01

Abbreviations: CTD, connective tissue disease; IPF, idiopathic pulmonary fibrosis; IPAF, interstitial pneumonia with autoimmune features; KL-6, Krebs von den Lungen-6; SP-D, surfactant protein D; PaO_2_, partial pressure of arterial oxygen; PaCO_2_, partial pressure of carbon dioxide in arterial blood; SPO_2_, saturation of percutaneous oxygen; FVC, forced vital capacity; Dlco, diffusing capacity of carbon monoxide; BAL, bronchoalveolar lavage; MΦ, macrophages; Ly, lymphocytes; Neut, neutrophils; Eo, eosinophils.

**Table 3:** The scores for the case from evaluators.

Score	A	B	C	D
FF	0	1	1	1
SMH	0	1	1	2
CIP	2	1	1	2
DPVC	0	0	1	0
Fat	0	0	1	0
Plasm	3	2	2	3
LyGC	1	2	1	3
AF	0	0	1	0

A–D: pathologists. FF: fibroblastic focus; SMH: smooth muscle hyperplasia; CIP: cellular interstitial pneumonia; DPVC: dense perivascular collagen; Plasm: plasmacytic infiltration; LyGC: lymphoid follicle with germinal center; AF: airspace fibrin.

## Data Availability

The data presented in this study may be made available from the corresponding author on reasonable request, subject to approval by the Ethics Committee of Nagasaki University Hospital.

## Supplementary Materials

The following are available online at https://www.mdpi.com/article/10.3390/diagnostics11081359/s1, Table S1: Patient’s distribution with diagnostic results for each evaluator.
