# Supplementary material for: Recognition of Connective Tissue Disease-Related Interstitial Pneumonia Based on Histological Score—A Validation Study of an Online Diagnostic Decision Support Tool
Source: Diagnostics (Basel). 2021 Jul 28;11(8):1359. doi: 10.3390/diagnostics11081359 (PMC8394442; doi:10.3390/diagnostics11081359)
Supplement: Supplementary file 1 [file diagnostics-11-01359-s001.zip › diagnostics-1293330-supplementary.pdf]

Validation study of pathological image analysis to extract autoimmune related groups from Idiopathic pulmonary fibrosis

Mutsumi Ozasa[1,2], Yoshiaki Zaizen[1], Kazuhiro Tabata[1], Kensuke Kataoka[3], Shuntaro Sato[4], Andrey Bychkov[1,5], Noriho Sakamoto[2], Hiroshi Mukae[2] ,Yasuhiro Kondoh[3], Junya Fukuoka[1,5*]

[1]Department of Pathology, Nagasaki University Graduate School of Biomedical Sciences
[2]Department of Respiratory Medicine, Nagasaki University Graduate School of Biomedical Sciences
[3]Department of Respiratory Medicine and Allergy, Tosei General Hospital
[4]Clinical Research Center, Nagasaki University Hospital
[4]Department of Pathology, Kameda Medical Center

*Corresponding author
Junya Fukuoka, MD, PhD
Department of Pathology, Nagasaki University Graduate School of Biomedical Sciences, Sakamoto, 852-8501, Japan

Table S1. Patient's distribution with diagnostic results for each evaluator.

Observer A				Observer B			
**Variable**	**CTD**	**IPF**	**P-value**	Variable	CTD	IPF	P-value
Sex			<0.01	Sex			0.03
Female	21	10		Female	15	16	
Male	10	53		Male	6	57	
Smoking history			<0.01	Smoking history			<0.01
Ex	8	49		Ex	5	52	
Never	23	14		Never	16	21	
CTD symptom			<0.01	CTD symptom			0.02
positive	19	18		Positive	13	24	
negative	12	45		Negative	8	49	
Autoantibody			<0.01	Autoantibody			<0.01
positive	23	27		positive	17	33	
negative	8	36		negative	4	40	
IPAF serological domain			<0.01	IPAF serological domain			0.03
positive	19	8		positive	14	13	
negative	12	55		negative	7	60	
CD4/8	1.61±1.38	3.09±2.45	<0.01	CD4/8	1.55±1.26	2.9±2.39	0.01

Observer C				Observer D			
**Variable**	**CTD**	**IPF**	**P-value**	**Variable**	**CTD**	**IPF**	**P-value**
Sex			<0.01	Sex			<0.01
Female	16	15		Female	18	13	
Male	9	54		Male	6	57	
Smoking history			<0.01	Smoking history			0.01
Ex	7	50		Ex	5	52	
Never	18	19		Never	19	18	
CTD symptom				CTD symptom			<0.01
positive	14	23	0.05	positive	16	21	
negative	11	46		negative	8	49	
Autoantibody			<0.01	Autoantibody			<0.01
positive	19	31		positive	19	31	
negative	6	38		negative	5	39	
IPAF serological domain			<0.01	IPAF serological domain			0.02
positive	13	14		positive	16	11	
negative	12	55		negative	8	59	
CD4/8	1.61±1.36	2.96±2.41	0.01	CD4/8	1.52±1.37	2.97±2.38	<0.01
